# Clinical efficacy of an antibody-based detection system for human papilloma virus infection in oral squamous cell carcinoma

**DOI:** 10.1007/s00784-020-03601-0

**Published:** 2020-10-23

**Authors:** Sebastian Blatt, Andreas Pabst, Stefanie Zimmer, Christian Walter, Bilal Al-Nawas, Maximilian Krüger

**Affiliations:** 1 Department of Oral- and Maxillofacial Surgery - Plastic Surgery, University Medical Centre, Augustusplatz 2, 55131 Mainz, Germany; 2 Department of Oral- and Maxillofacial Surgery, Federal Armed Forces Hospital, Rübenacher Straße 170, 56072 Koblenz, Germany; 3 Department of Pathology, University Medical Centre, Langenbeckstraße 1, 55131 Mainz, Germany; 4 Oral- and Maxillofacial Surgery - Facial Plastic Surgery, Mediplus Clinic, Haifa-Allee 20, 55128 Mainz, Germany

**Keywords:** Oral cancer, Head and neck cancer, Human papilloma virus, HPV, p16 expression, Biomarker

## Abstract

**Objectives:**

There is an increasing number of oral squamous cell carcinoma (OSCC) associated with HPV-16. However, p16 expression by immunohistochemistry as the current gold standard for a surrogate marker for virus infection reveals unsatisfying diagnostic accuracy. The aim of this study was to investigate a new rapid test for L1 antibody detection (Prevocheck®) and to validate its diagnostic performance.

**Materials and methods:**

In a prospective study, the HPV 16 association of all consecutive patients with an OSCC treated between 2015 and 2019 were analyzed by L1 seropositivity (via PrevoCheck®), p16 immunostaining, and partly multiplex PCR for subtype analysis.

**Results:**

Overall (*n* = 107), p16 expression was positive in 17 cases (15.9%), and L1 antibody seropositivity in 7 cases (6.5%). In PCR analysis, two cases of HPV35 and 50 were found. Total HPV prevalence was 8.4% overall and 6.5% for HPV-16. An inferior diagnostic accuracy for HPV-16-associated OSCC in comparison to PrevoCheck® was revealed.

**Conclusion:**

The rapid test for L1 antibodies showed an optimal sensitivity and specificity, positive and negative predictive value, and an overall diagnostic accuracy of 100%. However, HPV prevalence seems low in OSCC.

**Clinical relevance:**

L1 rapid test may represent an additional diagnostic staging method to detect HPV-16 association rather than p16 immunohistochemistry.

## Introduction

In head and neck oncology, oral squamous cell carcinoma (OSCC) is still an urgent health threat with a growing morbidity in various countries [1, 2]. While the incidence of OSCC related to the main risk factors tobacco and alcohol consumption is decreasing, there is an increasing number of OSCC associated with human papilloma virus (HPV), more precisely to the high risk type HPV 16 [3]. While many older studies reported a higher prevalence of HPV infection in OSCC (up to 82%) without differentiation between oropharyngeal squamous cell carcinoma (OPSCC) and OSCC, more recent studies revealed a lower overall prevalence of HPV infection in SCC located in the oral cavity of about 7% [4–7].

However, Gillison et al. reported of different profiles of HPV 16-positive and HPV 16-negative OSCC, and other studies confirmed that each profile should be considered as a distinct cancer entity [8, 9]. In contrast to HPV 16-negative OSCC, patients with HPV 16-driven OSCC are usually younger and show a good survival, with a 5-year survival rate of 82% and a low recurrence risk [10, 11]. The differential clinical outcome with potential therapy alterations led to an update of the TNM classification in the UICC 8th edition of the Union for International Cancer Control/American Joint Committee on Cancer for oropharyngeal carcinoma, where HPV status was implemented [12].

Today, several methods for HPV detection are frequently used in the clinical setting, with detection of HPV E6/E7 RNA expression, indicating active viral oncogene transcription in tumor cells, as the most accurate testing method [13–15]. However, because RNA isolation requires additional sample preparation steps and a larger number of tumor cells, the most widely used assay is the cyclin-dependent kinase inhibitor 2A oncoprotein (p16) expression by immunohistochemistry (IHC) staining, which displays a surrogate marker of oncogenic HPV infection, mainly for OPSCC [16]. In OSCC, where HPV association is considered less frequently, p16 IHC compared with high-risk HPV E6/7 RNA expression was shown as a poor surrogate biomarker of HPV infection, but, without valid alternatives, it is widely used as a cost effective surrogate marker and can be seen as the gold standard in clinical workflow for detection of HPV status [7, 12]. Since p16 IHC is relatively straightforward to interpret alongside the aforementioned benefits, the American Joint Committee on Cancer introduced p16 IHC to define HPV status in OPSCC [17].

However, in some cases, HPV infection could be an innocent bystander, and p16 independently positive, diagnostic improvements are much needed to avoid potential undertreatment in HPV positive but non-HPV-driven HNSCC [18].

While the early viral genes E1, E2, E3, E4, E5, E6, and E7 are necessary to control viral transcription, replication, and cellular transformation, the late genes L1 and L2 encode for the correspondent proteins for the capsid formation [19]. Recent studies have focused on the kinetics of HPV 16 serum antibodies against these viral proteins during treatment of HPV 16-driven OSCC [20]. To analyze antibodies to the major capsid protein HPV 16 L1, a rapid test (PrevoCheck**®** [Abviris, Germany GmbH]) is now available. While the test is easy to perform chair-side and delivers immediate results without any laboratory effort, its clinical benefit for the diagnosis of HPV status in OSCC should be further analyzed. Therefore, the aim of this study was to evaluate PrevoCheck**®** as a potentially reliable diagnostic tool for HPV status of OSCC in comparison with the p16 expression as the commonly used surrogate marker.

## Material and methods

### Patients

All subsequent patients (*n* = 107) included in the study were treated at the Department of Oral- and Maxillofacial Surgery, University Medical Centre Mainz, Germany, from November 2015 to August 2019. The inclusion criteria to participate in the study were the histopathological diagnosis of OSCC located between the lips and palatoglossal arch, including tongue, mandible, maxilla, and planum buccale. OSCC relapse, any other SCC localization, or other malignant tumor entities were defined as exclusion criteria. For all patients, HPV status, age, gender, tumor localization, TNM classification, adjuvant therapy, and clinical outcome were evaluated. All procedures performed within this study were in accordance with the ethical standards of the institutional research committee (“Ärztekammer Rheinland Pfalz, reference number: 837.223.15 (9991)”) and with the 1964 Helsinki declaration and its later amendments or comparable ethical standards. Informed consent was given by each participant in this study.

### HPV testing using PrevoCheck®

Every patient included in this study with a new diagnosis of OSCC was tested for HPV status before surgical treatment using PrevoCheck® (Abviris GmbH, Ammersbek Germany) according to the manufacturer’s protocol. For this, a venous blood sample was taken. All supplements and reagents were used at room temperature (15–30 °C). Twenty-five microliters of venous blood were added to the falcon tube with HPV-reagent, included in the kit, mixed by pipetting, and incubated for 10 min. Then 100 μl of the incubated blood sample were pipetted in the test-cartridge. After 10–15 min, the result (displayed by color changing or no color changing at the test line) was recorded for further analysis.

### Immunohistochemical HPV testing via p16 expression

**Fig. 1:**
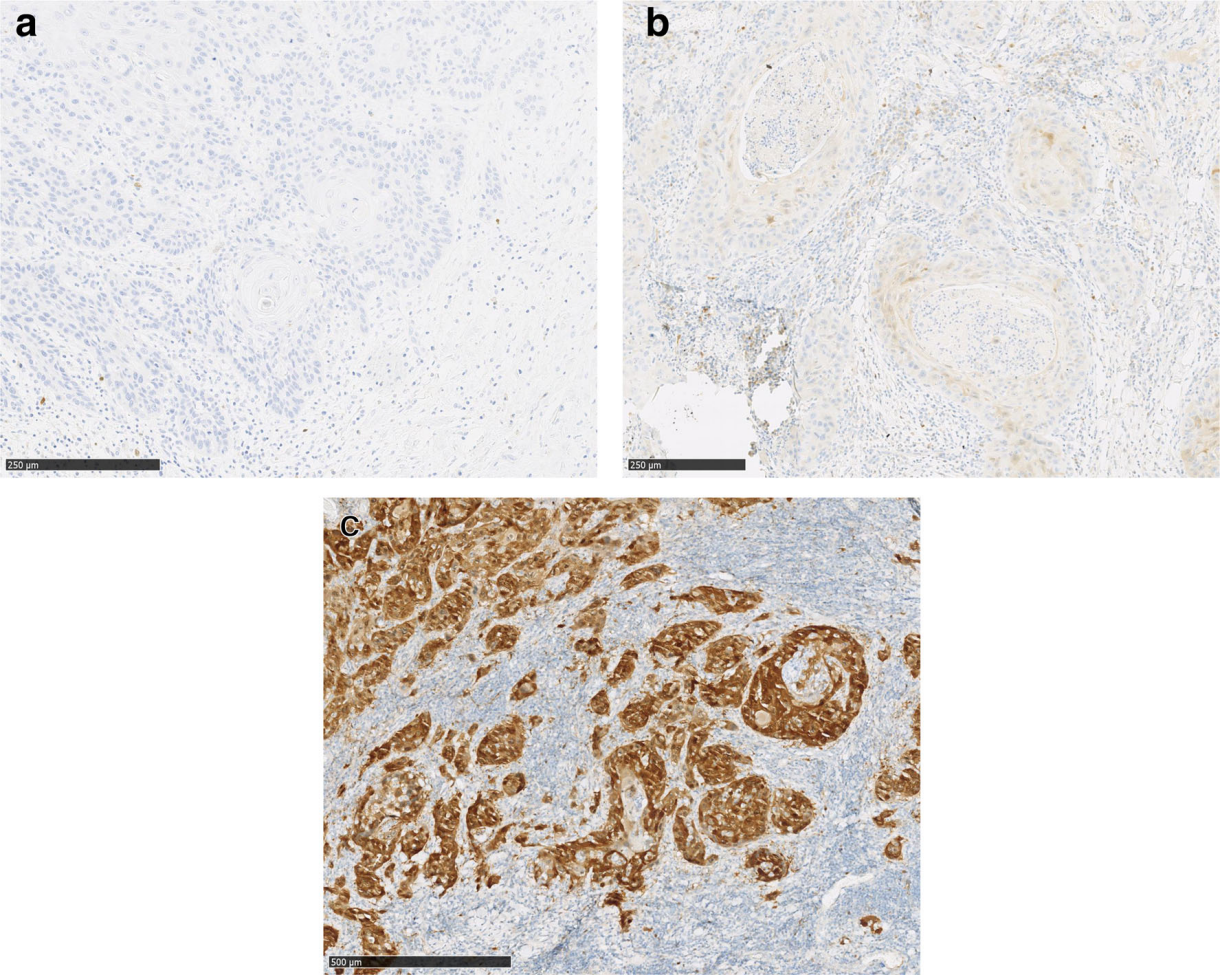
Exemplary samples for a negative (**a**), weak (**b**), and a strong (**c**) p16 IHC expression

After surgical removal of the OSCC, the resection specimens were analyzed by the Department of Pathology, University Medical Centre Mainz. HPV status was investigated by immunohistochemical staining against p16 as previously described [21]. Briefly, a p16 mouse monoclonal antibody (predilute, mtm-CINtech, E6H4) was used for IHC to determine p16 expression. A cutoff value of strong nuclear and cytoplasmic staining in ≥ 70% was defined as p16 positive, as recommended by the HNSCC guideline by the Federal Association of German Pathologists (please refer to exemplary cases of the cohort presented in Fig. 1a–c) [22]. A blinded pathologist independently performed the analysis.

### Reference HPV typing for inhomogeneous results

In the event of p16-positivity by IHC but L1 seronegativity (*n* = 10), an additional reference analysis was used for further HPV typing at the Institute for Pathology, University Medical Center Halle, Germany, as described previously [23]. In brief, a commercially available PCR-Multiplex kit (VisionArray HPV primer Kit 2.0, Zytovision, Germany) was used according to the manufacturer’s instructions. The kit tested for all known 41 HPV subtypes in order to detect a false negate p16 expression. The pathologist analyzing the data was blinded to the IHC results obtained before.

### Statistical analyses

Statistical analyses were performed using SPSS version 23 (IBM Deutschland GmbH, Ehningen, Gemany). 2 × 2 cross-tabulation was used for specificity, sensitivity, positive predictive value (PPV), and negative predictive value (NPV). For multivariate analysis, ANOVA testing was performed to detect possible risk factors for tumor relapse and overall survival. For graphic display, Kaplan–Meier plots were chosen in combination with log-rank Mantel–Cox regression. The Spearman correlation coefficient was taken to identify correlations of a positive test result and an HPV infection status. *P* values ≤ 0.05 were considered to be significant.

## Results

### Clinical characteristics of the patients

In total, 107 patients were included in the study. The mean age was 62 ± 14 years (standard deviation); 62% of the patients were male (*n* = 66) and 38% female (*n* = 41). Sixty-five percent of the malignancies were detected at an early tumor stage (T1/T2, *n* = 70). Twenty-eight percent (*n* = 30) initially showed one or more cervical lymph node metastasis. The most affected anatomical sites were the tongue (*n* = 38, 36%) and the alveolar ridge/gum of the mandible (*n* = 26, 24%), followed by the floor of the mouth (*n* = 22, 21%). Tumors of the alveolar ridge/gum of the maxilla (*n* = 9, 8%), and the cheek (*n* = 12, 11%) were sparse. No distant metastases could be detected. Histopathologically, the majority of tumors were categorized as well differentiated (G1/2: 86%, *n* = 92) (Table 1).

**Table 1:** Clinical characteristics of the patients

	All patients (*n* = 107)
Age (years ± SD)	62 ± 14
Gender	
Female	38% (*n* = 41)
Male	62% (*n* = 66)
Anatomical site of tumor manifestation	
Tongue	36% (*n* = 38)
Alveolar ridge/gum of the mandible	24% (*n* = 26)
Floor of the mouth	21% (*n* = 22)
Alveolar ridge/gum of the maxilla	8% (*n* = 9)
Cheek	11% (*n* = 12)
T-stage	
T1–2	65% (*n* = 70)
T3–4	35% (*n* = 37)
N-stage	
N0	72% (*n* = 77)
N+	28% (*n* = 30)
G-Stage	
G1/2	86% (*n* = 92)
G3	14% (*n* = 15)
Adjuvant therapy	
No therapy	54% (*n* = 58)
Radiation	25% (*n* = 27)
Radiochemotherapy	21% (*n* = 22)
Relapse	
No relapse	83% (*n* = 89)
Local recurrence	10% (*n* = 10)
Lymph node metastasis	7% (*n* = 8)
Tumor-associated death	13% (*n* = 14)

### Follow-up and patient outcomes

For primary therapy, all patients received surgical procedures in accordance with current guideline recommendations. In approximately half of the cases, no adjuvant therapy was needed (54%, *n* = 58). Due to advanced T-status and/or positive lymph node metastasis, 27 patients received radiation therapy (25%), in 15% in combination with cisplatin as radiosensitizer (*n* = 16). In the median follow-up interval of 36.6 ± 13.6 months, 10 patients developed a local recurrence (9%) and 8 patients a subsequent lymph node metastasis (7.5%). A total of 14 patients died (13%) due to their tumor manifestation (Table 1).

Advanced tumor size (T3/T4, *p* = 0.018) did significantly worsen overall survival. There was no significant association of L1 seropositivity (*p* = 0.186), a negative p16 expression (*p* = 0.360), tumor recurrence (*p* = 0.586), differentiation grad (*p* = 0.157), or lymph node metastasis (*p* = 0.119) on overall survival. In multivariate analysis, the age of the patients had a significant influence on overall survival (*p* = 0.019), while gender did not (*p* = 0.291).

### Prevalence of HPV infection

Overall, p16 expression was found to be positive in 17 tumor specimens (15.9%). L1 antibody seropositivity was found in seven cases (6.5%). The additional PCR analysis in ten cases where p16 expression was evaluated as positive and L1 serum antibodies were negative revealed an HPV infection with HPV subtypes 35 and 50 in two cases. For the remaining eight OSCC of this subanalysis, no HPV DNA could be detected by PCR. Therefore, the total HPV prevalence was found to be low at 8.4%, and an even lower HPV-16-associated infection rate of 6.5%.

### Sensitivity/specificity/diagnostic accuracy of PrevoCheck® vs. p16 expression

For p16 expression by IHC to detect HPV 16 association in OSCC, a sensitivity of 100% (95% confidence interval (CI): 59.04–100%), specificity of 90% (95%CI: 82.38–95.10%) and an overall diagnostic accuracy of 90.65% (95%CI: 83.48–95.43%) were demonstrated. Furthermore, a positive predictive value (PPV) of 41.18% (95%CI: 28–55.76%) and a negative predictive value (NPV) of 100% were found. PrevoCheck® revealed a sensitivity of 100%, a specificity of 100%, a PPV of 100%, and a NPV of 100% as well as overall diagnostic accuracy of 100%. A positive result in both methods was statistically significantly correlated with HPV-16 infection (*p* < 0.005).

## Discussion

This study is the first to exclusively investigate the HPV status of OSCC (*n* = 107) by L1 seropositivity (via PrevoCheck®), p16 immunostaining, and partly multiplex PCR. As a major result of this study, a low overall prevalence of HPV infection in patients with OSCC of 8.4% (6.5% for HPV 16) was revealed. Furthermore, it was shown that the immunohistochemical expression of surrogate marker p16 reveals a poor diagnostic accuracy for HPV 16-associated OSCC (90.65%). For the first time, a rapid test to analyze antibodies to the major capsid protein HPV 16 L1 was introduced. Here, an optimal sensitivity and specificity could be evaluated.

Since HPV-driven tumors can be seen as a distinct cancer entity and p16 status is embedded as an important prognostic marker in the current UICC classification, determination of HPV status in OSCC is crucial [12]. P16 expression was only associated with a tendency for improved overall survival in the investigated cohort. This is in accordance with the literature where a significant influence was found [24, 25]. Unlike Prevocheck®, staining was also positive for the subtypes HPV 35 and 50. However, mainly HPV 16 is associated with an increased OSCC risk rather than other subtypes [26]. The minor diagnostic accuracy is also in accordance with the literature, where in contrast to OPSCC multiple studies demonstrate that nonviral mechanisms are responsible for the majority of IHC p16 overexpression in OSCC [27]. In general, “classic” risk factors for OSCC are alcohol, tobacco, and especially in synergistic combination as well as poor oral hygiene [28].

Since a very high false-positive rate of p16 in OSCC could be demonstrated, this suggests it should not be used as a surrogate marker [29]. In a large retrospective study, D’Souza et al. found p16 IHC with a low specificity for HPV16 DNA in situ hybridization in nonoropharyngeal HNSCC. Therefore the authors concluded that “elevated levels of p16 may reflect the biologic characteristics of the tumor itself rather than the HPV status” [30]. Similar results were presented by Sgaramella et al. for OSCC of the tongue: although no HPV16 DNA was found, one-third showed p16 staining [31]. In contrast, Ndiaye et al. presented a percent positivity of p16 (INK4a) positive cases in HPV-positive oropharyngeal cancer cases of 86.7% [32]. In summary, the obtained results in combination with the literature question the use of p16 expression by IHC as the current gold standard for evaluation of HPV 16 status in OSCC despite its prognostic value on overall survival [16]. Furthermore, a critical application of TNM 8 is useful in the clinical setting as “the perception of TNM 8 as having therapeutic intention may lead to deescalating treatment regimens in p16-positive cases” [33].

Recent studies have evaluated the diagnostic impact of antibodies against the oncoproteins E6 and E7, the regulatory proteins E1 and E2, and the major capsid protein L1 by bead-based multiplex serology in the field of HNSCC as a screening method and/or for therapy control and outcome evaluation [14, 34]. For the subset of HNSCC located outside the oropharynx, including the oral cavity, HPV-16 E6 seropositivity had a high diagnostic accuracy of 97%, while L1 seropositivity had a lower diagnostic accuracy of 81%. Therefore, HPV-16 E6 seropositivity appears to be a highly reliable diagnostic marker for HPV-16-driven OPSCC [20, 35–37]. These results are thwarted with the cohort analyzed in this study showing an optimal diagnostic accuracy of the HPV-16 L1 antibody test for HPV-16 determination. In this study, L1 seropositivity had no influence on patient outcomes.

HPV diagnostics can be challenging. Firstly, the expression of L1 could be interpreted as being a consequence of a transient productive HPV-infection and not a malignant or premalignant transformation [38]. A higher expression of E6/E7 and a decreased L1 expression can be detected while the infection is transient. After the transient becomes a transforming infection, this is no longer the case [39]. However, detection of L1 does not exclude malignant transformation, due to the possibility of a synchronous appearance of transient and transforming infection [40]. Finally, not every infection with HPV 16 has to result in oral cancer but can be coincidental, so the real impact of a detected HPV infection cannot be estimated [41].

This study suffers from some major limitations. Firstly, with a low prevalence of HPV infection in the presented study collective, the diagnostic accuracy of PrevoCheck® cannot yet be generally evaluated. In addition, the eligibility of PrevoCheck® was not evaluated as a tool for postoperative monitoring for HPV-16-driven relapse. Furthermore, no negative (healthy) controls were analyzed. This could have helped determine test results in context of a coincidental infection. Furthermore, a HPV vaccination that may lead to false positive serological findings was not applied as an exclusion criterion. Finally, the multiplex PCR analysis only analyzed the subgroup that differed in p16 IHC and L1 seroprevalence. For an ideal verification of HPV association, the PCR analysis should have been run in addition to the p16 staining for all obtained samples.

## Conclusion

With an optimal diagnostic accuracy, the L1 antibody rapid test (PrevoCheck®) was superior to the commonly used p16 expression by IHC as a surrogate marker for HPV 16 association with OSCC. In addition, the eligibility of PrevoCheck® as a possible screening tool for HPV infection or for the early detection of relapse in HPV 16-positive HNSCCs cannot be evaluated yet. In accordance with previous studies, the present study showed a low prevalence (8.4% for all HPV subtypes, 6.5% for HPV 16) of HPV infection in SCC of the oral cavity. Therefore, in contrast to OPSCC, HPV association does not seem to play a major role in the carcinogenesis of OSCC. To date, it can be concluded that the test is not suitable as a screening or therapy monitoring tool for OSCC.
